# Interaction of Scientific Knowledge and Implementation of the Multilateral Environment Agreements in Relation to Digital Sequence Information on Genetic Resources

**DOI:** 10.3389/fgene.2020.01028

**Published:** 2020-09-16

**Authors:** Kunihiko Kobayashi, Eiji Domon, Kazuo N. Watanabe

**Affiliations:** ^1^Research Institute for Humanity and Nature, Kyoto, Japan; ^2^National Agriculture and Food Research Organization (NARO), Tsukuba, Japan; ^3^Faculty of Life and Environmental Sciences, University of Tsukuba, Tsukuba, Japan

**Keywords:** digital sequence information on genetic resources, multilateral environment agreements, scientific knowledge, genetic sequence data, Convention on Biodiversity (CBD), International Treaty on Plant Genetic Resources for Food and Agriculture (ITPGR)

## Abstract

Integration of scientific knowledge into negotiations of the Multilateral Environment Agreements (MEAs) is crucial to effective implementation of those MEAs by ensuring uniformity in their terminology. Recent innovations in the field of biotechnology provoked a discussion over “Digital Sequence Information” (DSI) in fora of several MEAs. In the context of this discussion, the term DSI remains ambiguous and encompasses a wide range of concepts, including, at least, DNA/RNA base sequence data. We focused on how the term “DSI” was regarded in negotiations of the Convention on Biological Diversity and the International Treaty on Plant Genetic Resources for Food and Agriculture, analyzed the changes of terminology for DSI in the opinions or views of the Parties in the supreme decision-making bodies of these agreements from the perspective of the MEAs implementation. Based on these efforts we suggest the ways and means to support challenges regarding integration of scientific knowledge into MEAs.

## Introduction

**FIGURE 1:**
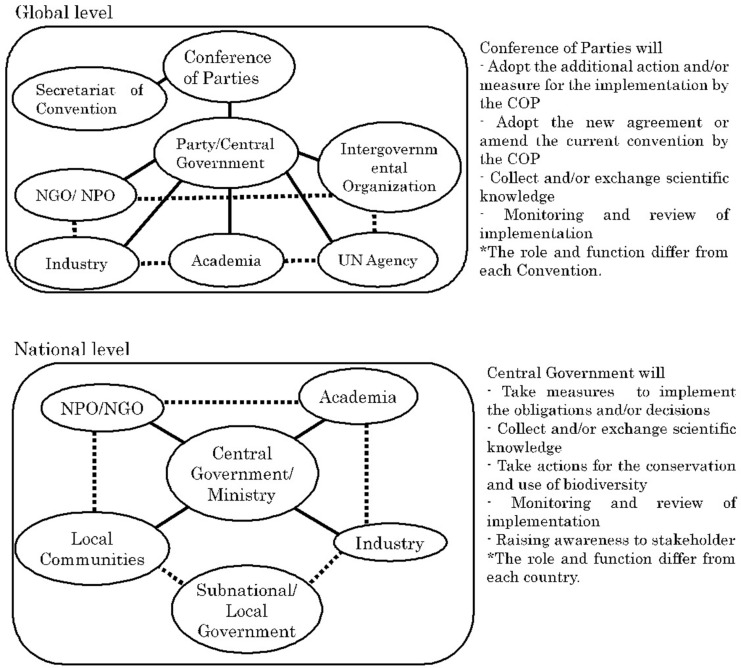
Interaction of scientific knowledge among various sector at the Global and National Levels.

Global environmental issues such as climate change and the loss of biodiversity are being addressed through the Multilateral Environment Agreements (“MEAs”) by nearly all countries. MEAs impose various obligations on each country to achieve the objectives of the agreement^1^. The obligations imposed by the Convention on Biological Diversity (CBD), for example, are implemented within countries by legislative measure (for example, law), administrative measure (for example, regulation and guideline), and/or policy measure (for example, public awareness). While it is important to identify the phenomena and causes of the phenomena in order to effectively address such measures, the CBD recognizes the general lack of information and knowledge on biodiversity and oblige Parties to promote the exchange of scientific knowledge and technology or scientific cooperation. However, such scientific knowledge is generated by the United Nations (“UN”) Agency, Industry, Intergovernmental Organization, Non-Governmental Organizations/Non-Profit Organizations, and academia such as universities and research institutes. Therefore, without the cooperation of such sectors, the central government as a Party with decision-making authority cannot achieve the objectives and implementation of the obligation, of CBD. Scientific knowledge generated by various sectors not only interacts at the national and global levels directly and/or indirectly, but also between the national and global levels (Figure 1). With regard to the relations with other MEAs, at the global level they may also interact between MEAs as they may be indirectly affected in the process of coordination with the Secretariat of agreement as a UN Agency and coordination within the central government. However, it is up to the Party with final decision-making authority to decide whether such interactions will work or not. MEAs and scientific knowledge are interrelated in circumstances such as the following: (1) when scientific knowledge raises questions regarding the draft of an agreement (Glowka et al., 1994) (2) when Parties attempt to come to consensus on terms, definitions, scope, and actions regulated by the agreement, (3) when reconsideration of matters regulated by the agreement is warranted due to new knowledge or technologies (in some cases, this may escalate to circumstance 1), and (4) when implementation of the agreement requires scientific knowledge (e.g., for example, Article 6 of CBD and Article 5 of International Treaty on Plant Genetic Resources for Food and Agriculture). In other words, agreements require scientific knowledge not only during the drafting process but also throughout the process of implementation. Scientific knowledge refers to the objective knowledge accumulated by experts in the scientific community. Accumulating knowledge in this manner occurs in various academic fields; in particular, for global-scale environmental protection, the Intergovernmental Panel on Climate Change has been developed in the climate change field and platforms such as the representative Intergovernmental Science-Policy Platform on Biodiversity and Ecosystem Services are being built in the biodiversity field. Apart from these examples, scientific knowledge is expected to play a critical role from the agreements in various biodiversity related fields, and there is a trend toward making agreements on these (Mauerhofer, 2019; Nishimura, 2019).

In recent years there have been technological advancements and innovations in various fields, including artificial intelligence. In the field of biotechnology, these advances include genome editing and synthetic biology technologies that enable the artificial creation of, e.g., enzymes and cells. Concerns about the impact of such technological developments on the objectives of the CBD, in particular the third objective of fair and equitable sharing of the benefits arising out of the utilization of genetic resources, were raised by the African region at the 13th meeting of the Conference of the Parties held in Mexico in November 2016. With the advent of synthetic biology technologies, users of genetic resources can directly access publicly available sequences in databases such as GenBank, where they obtain the data from genetic resources while remaining exempt from benefit-sharing obligations (Kobayashi, 2017) instead of coordinating with the developing countries where the former are found. This issue, which applies to all genetic resources, was not only raised at the CBD but also by the governing body of the International Treaty on Plant Genetic Resources for Food and Agriculture (ITPGR)^2^, which regulates the use of plant genetic resources for food and agriculture (PGRFA), and the Pandemic Influenza Preparedness Framework (PIPF) of the World Health Organization (WHO) regarding the sharing of influenza viruses, access to vaccines, and other benefits. Unlike the CBD, which delegates regulation to the laws of each country and does not set substantive rights and obligations regarding the acquisition of genetic resources, the ITPGR established a Multilateral System (MLS) and Standard Material Transfer Agreement (SMTA) (Manzella, 2013). Although issues related to digital sequence information (DSI) on genetic resources were considered during the discussions on enhancing the functioning of the MLS in the ITPGR meeting, several Parties were opposed to the Chair’s proposal regarding the handling of DSI at the 8th session of the governing body held in November 2019, and no consensus was reached^3^. However, the PIPF stipulates that—in addition to the mechanism of global sharing of genetic resource of H5N1 and other influenza viruses with human pandemic potential—the framework should share genetic sequence data (GSD) obtained from viruses^4^. The PIPF defines genetic sequences as ‘the order of nucleotides found in a molecule of DNA or RNA. They contain the genetic information that determines the biological characteristics of an organism or virus.’ However, the handling of GSD including the benefit sharing in the context of PIPF was relegated to future discussions, and these PIP Advisory Group and PIP Review Group discussions were still in progress as of February 2020^5^. Ever since the handling of DSI was proposed at the 13th meeting of the Conference of the Parties to the CBD, related agreements seem to be becoming further integrated via information sharing between these agreements^6^. A common point of discussion at these international forums is the use of the provisional term ‘DSI.’ In the notes of the decision of the 13th meeting of the Conference of the Parties to the CBD, it is stated that the terminology for DSI is ‘subject to further discussion in the study and the expert group.’ Defining terminology not only benefits a single agreement, whether CBD as the umbrella Convention, ITPGR as a specialized ABS instrument or not, but also contributes to development of other international treaties discussing the same topic. Replacing and defining DSI with the appropriate term will also be advantageous for implementation of the agreement and can be an opportunity to incorporate scientific knowledge in the implementation process from the view of the international environment law.

In addition, as the terms GSD and DSI are used, each agreement has a system or a provision for data or information. Article 17 of ITPGR requires Parties to work together to develop the Global Information System on Plant Genetic Resources for Food and Agriculture (GLIS) and facilitate information exchange. Regarding the relationship with PIPF, the Global Initiative on Sharing All Influenza Data (GISAID) is an existing database, although it is not a system built under the framework. GISAID has initiated a mechanism to promote global sharing of all influenza virus data and to jointly publish results and is recognized as an essential mechanism for sharing influenza gene sequences and metadata^7^. GSD of new coronaviruses originating in China and occurring in various countries are also shared through GISAID^8^. Although the relationship between PIPF and GISAID is not necessarily the same as GLIS in ITPGR from a legal point of view, GISAID was developed in 2008 through discussions within the scientific community and negotiations with national governments around data sharing (Elbe and Buckland-Merrett, 2017). When considering alternative terms to the DSI, it is inevitable to discuss how to position the relationship with information sharing systems or existing databases established under the agreement.

To date, studies dealing with DSI can be broadly divided into those that address approaches to regulation and management of DSI acquisition and use (e.g., material transfer agreements, open access) and those that focus on DSI from a legal or policy-related perspective (Bagley et al., 2020). Common across these studies is the mention of what the term ‘DSI’ used in these negotiations refers to. Terminology used includes ‘natural information,’ ‘genetic resource information,’ and ‘genetic sequence data’; these terms were also mentioned in the report of the *Ad Hoc* Technical Expert Group (AHTEG) established in accordance with decision XIII/16 adopted at the Conference of the Parties to the CBD held in 2018. The discussion processes of the PIPF (the Intergovernmental Meeting on Pandemic Influenza Preparedness and the Open-Ended Working Group of Member States on Pandemic Influenza Preparedness) are still at the stage of discussing specific handling provisions using the term ‘GSD’; therefore, it cannot be confirmed whether there were opportunities to integrate scientific knowledge, such as through the submission of views or opinions from the government, relevant organizations, and stakeholders such as academia and industry. However, according to Gostin et al. (2014) and Hay and McCauley (2018), a wide range of Parties including government and public health officials, scientists and intellectual property experts, as well as GISRS and WHO, have participated in these discussions. Past research results have also identified the existence of uncertainties in the scientific knowledge (Dovers et al., 1996). However, the role expected of the AHTEG established under the CBD and other expert groups is to address the issue through the process of reducing and removing such uncertainties, while the Conference of the Parties to the CBD serves to unify knowledge and make decisions. In that sense, the Conference of the Parties is also a process of learning for governments. Therefore, the discussion of Conference of the Parties—and by extension, analysis of opinions by Parties to the CBD used as a basis for discussion—are necessary components for integrating scientific knowledge and policy, and it is possible to understand the intentions and strategies of each Party as to whether it intends to accept various types of knowledge and reflect them in policy decisions. In recent years, attention has been paid to the role of “uncertainty.” According to Knaggård (2014), the role is first, to the need to decrease uncertainty in order to enable policy-making (e.g., Shukla et al., 2009); second, to the need to communicate uncertainty to enable policy-making (e.g., Ascher, 2004; Kandlikar et al., 2005; Smith and Stern, 2011); and third, to policy strategies to cope with uncertainty (e.g., Mearns, 2010; Funke and Paetz, 2011; Smith and Stern, 2011). These three points do not seem to indicate a role but rather a step toward policy-making. Not many studies have analyzed how scientific knowledge is translated into actual discussions and negotiations in multilateral agreements on specific issues at these three stages (Choi et al., 2009). This is probably because it is difficult to grasp the position of each government in relation to the debate. However, in this study, it is possible to supplement it by the documents submitted by each government to the Secretariat in advance and the open negotiation meeting, and it is considered that the value as a substantive study can be found. Such research can also provide the scientific community with an overview of the roles expected of the practical research community.

The base sequence database referred in the statement by developing countries was established in 1980 and has been used by researchers and companies. Each database has promoted a policy of free acquisition and use^9^. However, it has been pointed out that the implementation of this policy also ignores issues related to inequalities in access and capacity to assess DSI (Aubry, 2019). In addition, if the discussion on the term DSI discussed in this study is not properly addressed, there is a possibility that uncertainty will remain in the future use of data, and there are concerns over its long-term impact. Therefore, it is expected that key stakeholders such as researchers and companies will actively participate in and contribute to the discussions. However, when actively participating, it is considered to be necessary to sort out the content to bridge the understanding and recognition of each Party.

In this study, we focused on international negotiations related to DSI/GSD in MEAs, analyzed the changes of terminology for DSI/GSD in the opinions or views of the Parties with decision-making authority in the supreme decision-making bodies from the perspective of implementation of MEAs, and suggested the way forward to solve the challenges in relation with integration of scientific knowledge.

## Research Purpose and Method

Using negotiations related to DSI/GSD as an example, this study examines the dynamics of multilateral environment agreements—in particular, discussions by the CBD and ITPGR—to analyze how each country accepts uncertainties in scientific knowledge and accommodates them into the implementation of the agreements, including efforts by information sharing systems under the agreements or existing databases. To identify the views expressed by each Party to the CBD and ITPGR, references are made to proposals submitted to the secretariat of the respective agreements and negotiation processes such as working groups and the Conferences of the Parties. For proposals submitted to the secretariats, we referenced responses to SCBD/SPS/DC/VN/KG/jh/86500^10^ and SCBD/NPU/DC/VN/KG/RKi/87804^11^, and NCP GB8 020 MYPoW/DSI^12^, and NCP GB8-016 MYPoW/DSI^13^. For negotiation processes such as working groups and the Conferences of Parties, we referenced the reports of each meeting, the Earth Negotiations Bulletin issued by the International Institute for Sustainable Development.

## History of CBD and ITPGR Discussion: Country-Level Views

Although both agreements continue to discuss handling of the term DSI, we analyzed the views expressed at the ITPGR and CBD separately as discussions were at different stages^14^. However, views submitted by each country (Table 1) are handled together because they were submitted as a country.

**TABLE 1:** Countries and regions that submitted a view.

**Country/Region**	**ITPGR (2018)**	**CBD (2017)**	**CBD (2019)**
African Group	×	✓	✓
Argentina	✓	No clear comment	✓
Australia	×	✓	✓
Belgium	✓	×	×
Belarus	×	No clear comment	No clear comment
Brazil	✓	✓	✓
Canada	✓	No clear comment	✓
Central African Republic	No clear comment	×	×
Colombia	×	×	✓
Costa Rica	×	×	No clear comment
Ecuador	×	✓	×
Eswatini	✓	×	×
Ethiopia	×	×	✓
European Union and its Member States	×	No clear comment	✓
Germany	✓	×	×
India	×	✓	✓
Italy	✓	×	×
Iran	×	×	✓
Japan	✓	✓	✓
Kuwait	✓	×	×
Madagascar	×	×	No clear comment
Mexico	×	No clear comment	✓
Namibia	No clear comment	×	×
Poland	✓	×	×
Republic of Korea	✓	×	✓
Spain	✓	×	×
South Africa	×	×	✓
Switzerland	×	✓	✓
the Netherlands	✓	×	×
Venezuela	×	No clear comment	×
The United Kingdom	✓	×	×
The United States	✓	✓	✓

*✓: clear response. ×: no response.*

### Country-Level Views on Terminology

The views of each country regarding the terminology are shown in Table 2. With some exceptions, there are two groups: those who insist on ‘natural information’ or ‘genetic information’ and those who insist on GSD or ‘nucleotide sequence data.’ The difference in terminology can be thought of as whether amino acid sequences or protein sequences are involved. In countries where the term GSD is proposed, it is stated on the basis that the term is defined in the PIPF and used in the scientific community (Belgium, Canada, the EU, Germany, Japan, Switzerland, the Netherlands, the United Kingdom, the United States, and South Korea). Differences can be seen in the terms proposed and the content in some countries. Argentina and Colombia, like other developed countries, have proposed the term genetic information, but the content is nucleotide sequences of DNA and RNA and does not include amino acid sequences or protein sequences. The reasons for this difference are not clear, but the scientific knowledge of the term differs from those of developed countries proposing the term GSD. In addition, Spain proposed a terminology different from other developed countries, including the EU and EU Member States, even though the subject under discussion is the nucleotide sequence of DNA or RNA. The same can be said of Italy. Although these cases are different, they may imply uncertainty about scientific knowledge in the discussion of what terms are appropriate.

**TABLE 2:** Views on terminology related to DSI.

**Country/Region**	**Nucleotide sequence of DNA/RNA**	**Sequence of Amino acid and/or protein**	**Terminology proposed or used in the submitted document**
African Group	✓	✓	NI, GI
Argentina	✓	×	GI
Belgium	✓	×	GSD
Brazil	✓	✓	GI
Canada	✓	×	GSD
Colombia	✓	×	GI
Ecuador	✓	✓	?
Eswatini	✓	✓	GSD
Ethiopia	✓	✓	GI, GSD
European Union and its Member States	✓	×	GSD
Germany	✓	×	GSD
India	✓	✓	GI, GSD
Italy	✓	✓	GSD
Iran	✓	✓	GI
Japan	✓	×	GSD
Mexico	✓	✓	?
Poland	✓	✓	GSD
Republic of Korea	✓	×	GSD
Spain	✓	×	Biological Database
South Africa	✓	✓	NI, GI, GSD
Switzerland	✓	×	GSD
The Netherlands	✓	×	GSD
United Kingdom	✓	×	GSD
United States	✓	×	GSD

*NI, Natural Information; GI, Genetic Information; GSD, Genetic Sequence Data.*

### Dynamics of Discussion in the CBD

The term DSI has not been discussed since the 13th Conference of the Parties to the CBD held in 2016. At the very least, the Conference of the Parties has called for further discussion as the term “DSI” is not appropriate. Therefore, only the implementation of the scoping study, provision of information from Parties, and consideration by AHTEG are being carried out in order to examine appropriate terminology. Therefore, there is no specific discussion in CBD, but this section only provides an overview of the accumulation of scientific knowledge in CBD.

In the decision (CBD/COP/DEC/XIII/16)^15^ adopted at the 13th Conference of the Parties in 2016, the terms of reference of AHTEG defines as to “identify the different types of digital sequence information on genetic resources that are relevant to the Convention and the Nagoya Protocol” and raised the following; (a) The nuclear acid sequence reads and the associated data; (b) Information on the sequence assembly, its annotation and genetic mapping. This information may describe whole, individual, or fragments of genes, barcodes, organ genes, or single nucleotide polymorphisms; (c) Information on gene expression; (d) Data on macromolecules and cellular metrics; (e) Information on ecological relations and biotic factors of the environment; (f) Function, such as behavioral data; (g) Structure, including moral data and phenotype; (h) Information related to taxonomy; and (i) Modalities of use.

According to ‘A Fact-Finding and Scoping Study on DSI on Genetic Resources in the Context of the CBD and the Nagoya Protocol’(Sarah and Wynberg, 2018) published in 2018, the term DSI is not used in the scientific community; the terms GSD, nucleotide sequence data, nucleotide sequence information, and genetic sequence information are used instead. This highlights that harmonizing terminology is difficult because these differences in terminology ‘reflect the differences in the material referred to, as well as the speed and transformative nature of technological change today.’ In the decision adopted at the 14th meeting of the Conference of the Parties to CBD^16^, it was decided that the AHTEG, which was entrusted with reviewing the study, would continue discussing the terminology due to its broad range of interpretations, as mentioned earlier. As the AHTEG is tasked with developing ‘options for operational terms and their implications to provide conceptual clarity on digital sequence information on genetic resources,’ an operational definition is expected to be decided at the 15th meeting of the Conference of the Parties. It should be noted that although a common consensus was reached—at the 14th meeting of the Conference of the Parties—that DSI was not an appropriate term, the Parties did not go so far as to discuss what type of terminology would be appropriate. A commissioned research report published in January 2020 (Wael et al., 2020) further considers the flow of data and information from genetic resources and proposes new logical groups for DSI subject matter, as well as evaluates alternative terminology for DSI and specifies priority issues that must be addressed to clarify the concept of DSI. Therefore, it does not refer to the appropriate term.

### Dynamics of Discussion in the ITPGR

Discussion of DSI in the ITPGR was handled at the seventh session of the governing body held in 2017. Handling of DSI was discussed from the perspective of whether it should be included in the agenda, and it was decided in Resolution 13/2017^17^ that the term would be considered in the Multi-Year Program of Work (MYPoW). In the resolution, the term DSI was used with the following note stating that the appropriate term is to be used in the future: ‘The term is taken from decision CBD COP XIII/16 and is subject to further discussion. There is a recognition that there are a multiplicity of terms that have been used in this area (including, *inter alia*, “genetic sequence data,” “genetic sequence information,” “genetic information,” “dematerialized genetic resources,” “*in silico* utilization,” etc.) and that further consideration is needed regarding the appropriate term or terms to be used.’ However, at the seventh session of the governing body, developing countries such as those in the African region argued that it should be included in ongoing negotiations on revisions to the SMTA. The same arguments were made at the eighth session of the *Ad Hoc* Open-ended Working Group to Enhance the Functioning of the Multilateral System of Access and Benefit-sharing (OWG-EFMLS) held in 2018. The terminology was then addressed at the ninth session of the OWG-EFMLS. Before the treaty Parties began negotiations, the Secretariat explained, ‘the term “GSD” or “information associated with PGRFA” may be more appropriate’^18^, and the Co-Chairs made the same proposal to the member countries of the Working Group^19^. The term GSD was suggested in the above-mentioned proposal due to several countries that had previously suggested using the term GSD^20^. However, when negotiations began at the ninth session of the OWG-EFMLS held in June 2019, several countries, developed countries in particular (North America and Europe), proposed using the term ‘information associated with PGRFA’ rather than the term GSD, which was proposed in the submissions. Their reasoning was that it was preferable to use language used in the Treaty^21^. Asia, Near East, and Africa responded that reference to information ‘generated from’ the material, rather than ‘information associated with PGRFA,’ would be more suitable for the issue under consideration. Despite this, ‘information associated with PGRFA’ was incorporated into the interim report (FAO, 2019).

Subsequently, the resumed ninth session of the OWG-EFMLS convened (October 2019) followed by the eighth session of the governing body where discussions were continued informally between the treaty members, during which no consensus was reached through negotiations on revisions to the SMTA and the MLS of the PGRFA, including handling of the term DSI. However, during the eighth session of the governing body, the chairperson of the governing body and the informal review meeting twice submitted proposals to serve as springboards for discussion. In the document stating the chairperson’s final proposal, the term GSD and its definition used in the PIPF were incorporated into the SMTA revision proposal.

As illustrated above, the issue with terminology began with the term DSI used by the CBD, and although North America, Europe, and Japan suggested the term GSD, other terms were proposed because of the reference to Treaty language. However, as was mentioned in the proposals submitted by North America, Europe, and Japan, the term GSD is used in the PIPF; not using this term while stating that Treaty language should be used will introduce a wide range of interpretations to the agreement while maintaining the complexities of the problem with the term DSI. Therefore, it is unlikely that the problem will be solved on a fundamental level. Because it is becoming increasingly difficult to distinguish between data and information (Wael et al., 2020), making such a concession is possible, but in any case, it appears that there was no compelling reason for solving the issue.

### Section Conclusion

The views of the countries, the views of the expert groups, and the terms proposed in the actual negotiations are considered to be fluid when analyzed in terms of the CBD and ITPGR. However, it appears that the terms proposed by governments and those discussed in the negotiations, as opposed to those proposed by the experts group such as AHTEG, are intended to be targeted. This indicates that, through the intermittent processes of AHTEG, Subsidiary Body and Conference of the Parties, in addition to DSI, terms used at the Conference of the Parties will be limited to two other terms, such as GSD. In other words, it can be said that this is evidence for reducing uncertainty. The function of the expert group is to review and comment on specific issues as delegated by the Conference of the Parties. Therefore, there will be no discussion unless the delegation from the Conference of the Parties includes an instruction to evaluate the term “GSD” and the term “information associated with PGRFA” used in ITPGR negotiations.

On the other hand, focusing on the discussions at the CBD, it has not been discussed at the Conference of the Parties, despite the fact that, in addition to the expert group, governments have proposed several terms. In order to reduce uncertainty, it is necessary to present a common understanding and strategy of the Conference of the Parties, as the role of the Conference of the Parties, which has been delegated to the Meeting of Experts, based on the opinions expressed at the Meeting of Experts, such as whether it is necessary to change terminology in accordance with technological development or whether terminology should be defined in a manner that takes technical development into account.

## Relationship With Information-Sharing Systems Under the Agreements or Existing Databases

Mechanisms for sharing information or data, such as GLIS and GISAID, are not limited to the environment, but are also found in treaties and institutions in various fields. GLIS, as described above, is an information sharing system defined by the ITPGR. At the 6th meeting of the Governing Body held in 2015, a work plan (Resolution 3/2015)^22^ was formulated. As long as the system is defined by the ITPGR, its operation would be entrusted to the Council, the highest decision-making body of the Agreement (Paragraph 3 of Article 19 of the ITPGR). The resolution of GLIS of the 8th Governing Body, in which negotiations on MLS function improvement broke down, states (Resolution 4/2019)^23^, indicating that discussions are being conducted in the context of GLIS. Some systems, such as GISAID, are not systems/databases established under the agreement but are related to the implementation of the agreement. Because cooperation is sometimes requested through the decisions of the Conference of the Parties, even if not under the control of the agreement, the modality of cooperation is carried out through various means. In this study, we analyze how GLIS and GISAID, which are managed under the agreement, are defined and addressed.

### How GLIS Works and What Information It Covers

GLIS is a database system for non-monetary benefit sharing based on Article 17 of ITPGR, and non-confidential information concerning PGRFA held by treaty parties is to be collected in GLIS and made publicly available through the Internet. The 6th Governing Body in 2015 adopted a work plan for the specific activities of GLIS over the period 2016–2022^24^, and since 2016, the Scientific Advisory Committee for GLIS (SAC-GLIS) has been convened by experts to discuss the design of databases, the format of data, and the types of data to be included biannually. The implementation and operation of the GLIS database is carried out by the ITPGR Secretariat and FAO’s Information Systems Division.

So far, the information on PGRFA contained in GLIS is limited to the range of passport data of the genetic resources identified by DOI in accordance with the results of the discussion in Part 2, SAC-GLIS (SAC-GLIS -2, 2017), in which each Party assigns Digital Object Identifiers (DOI) to each PGRFA included in MLS. At the third SAC-GLIS (SAC-GLIS -3, 2018), discussions were held on the propriety and significance of the provision of GSD on PGRFA (GSD-PGRFA) through GLIS and the ideal way to provide GSD-PGRFA regarding GSD with respect to PGRFA, in accordance with the consultation of the ITPGR Governing Body. As a result of the discussions, the Committee recognized that DOI is useful for linking individual genetic resources with GSD-PGRFA derived from them, and expressed the view that, given the current status of GLIS development, it is useful to include GLIS in the main sequence database such as International Nucleotide Sequence Database Collaboration (INSDC)^25^. In connection with the SAC-GLIS-3 report, the 8th Governing Body in 2019 adopted acknowledgments for those who have provided DOI-related phenotypes or “DSI/GSD” information^26^.

Thus, in consideration of the development status of the system, GLIS is expected to include not GSD itself but a unique accession number that is assumed for each sequence in major international nucleotide sequence information databases as representative data of DSI/GSD.

### How GISAID Works and What Information It Covers

GISAID Database Access Agreements (DAA) govern the access and use of data registered with GISAID^27^. The key features of DAA are: (1) Encouraging data sharing by protecting the ownership of data providers and requiring the approval of those who provide samples and create data; and (2) No restrictions are placed on the use of data by registered users who participate in DAA (Shu and McCauley, 2017). In DAA, the data are as follows.

(i) Sequence data and other associated data and information contained in the GISAID EpiFlu^TM^ Database pertaining to influenza viruses.
(ii) Any annotations, corrections, updates, modifications, improvements, derivatives, or other enhancements to any such data contained in the GISAID EpiFlu^TM^ Database.
(iii) Any safety information relevant to the use of the data or regulatory approval of vaccines or other therapies that embody or utilize the data contained in the GISAID EpiFlu^TM^ Database.

The data are divided into three categories, but the “data” used here is centered around array data. The DAA does not define sequence data, but documents submitted to the CBD Secretariat and papers on the system describe it as a GSD^28^. In addition to the GSD, information such as date of sample collection, source of sample, date of virus collection, and antiviral drug sensitivity can be included (Elbe and Buckland-Merrett, 2017). The sequence data are complemented from INSDC.

### Section Conclusion

GLIS and GISAID treat GSD as one of the data and information related to viruses and plant genetic resources. The difference between the two systems is whether contracts need to be exchanged and whether there are arrangements for subsequent use. The difference is that there are two types of data: data for which there are explicit arrangements for subsequent use, and data for which there are no such arrangements. The fact that data are handled differently by different databases can be cumbersome in the big data era. Given this background, there seems to be some rationale for European countries, in particular, to try to build a multilateral system. On the other hand, both databases and systems are complementary to INSDC. Therefore, it can be seen that a single database is not independent but is based on reciprocity.

## Discussion on the Implementation of the Agreement, Including Terminology, From the Perspective of Uncertainty

A review of the arguments and processes of each country in the CBD and ITPGR suggests that the terms proposed at the CBD expert meetings are subject to trial and error through the Conference of the Parties to the CBD and the OWG-EFMLS and the Governing Body in the ITPGR. However, as seen in the GLIS resolution, the DSI and the GSD were finally adopted in parallel. It indicates that the resolution adopted in this way may not be effective in terms of the implementation, because there is no common understanding or interpretation of the terminology and, as a result, each Party has its own interpretation (Brink and van Hintum, 2020). Although there has been little discussion of specific terminology in the nearly 30-year history of CBD, for example, Party discussed whether to replace the term “indigenous and local communities” in the text of the CBD with the term “indigenous peoples and local communities” in the documents used in the future document of the CBD including the decision of the Conference of Parties without amendment of Convention. As a result of the negotiation, Conference of Parties decided to use the term “indigenous peoples and local communities” with a variety of annotations such as “as appropriate”^29^. However, Canada unilaterally declared in the plenary at the closing session of the 12th Conference of Parties that it would not consider the decision in implementing the obligations under the CBD^30^. In the absence of a compliance mechanism in the CBD, the challenges arise as to how each Party will incorporate the decisions adopted by the Conference of the Parties in addition to the national implementation of the obligations imposed by the CBD. The “voluntary peer-review mechanism (VPM)” currently under consideration for the post- 2020 target under the CBD can contribute to addressing these issues. To date, each Party has prepared and submitted a national report as an obligation of the CBD. However, the report was only used as a primary source for the Global Biodiversity Outlook. Since the VPM provides feedback to the Party directly, it is not a traditional compliance mechanism, but in a sense it may be capable of functioning as a compliance mechanism (Ana et al., 2018).

Regarding ITPGR, considering the use of the terms DSI and GSD in the GLIS resolution as described above, it can be assessed that decisions were made in accordance with scientific knowledge. However, it should be noted that this means acceptance of scientific knowledge based on the agreement, but it does not mean that countries, especially developing countries, have accepted scientific knowledge^31^. This is because the issue of terminology became a political issue. The fact that the analysis of documents submitted by each country listed GI instead of GSD as including amino acid sequences and protein sequences is also a sign that they expect more profit sharing. This trend is observed not only in the DSI but also in the negotiation process of the Nagoya Protocol^32^ and ABS national legislation^33^. It is recognized that the acceptance of scientific knowledge will define the scope of the treaty, i.e., ensure transparency and accountability, and effectively facilitate its implementation. Therefore, the use of (scientifically unacceptable) terms that are difficult to ensure objectivity may provide various interpretations to the operation of the agreement. As one of the solution to avoid this, the decision-making Parties are required to base their decisions on scientific knowledge, and it is necessary to ensure the independence of the researchers or research communities that support them. In addition, this paper focuses on CBD and ITPGR in relation to the discussion on the term DSI, but the common issues seem to derive from and develop dynamically not only one agreement but also related agreements. However, in order to confirm whether such a development has been made strategically or not, it is necessary to confirm the following two points. While CBD apply to all genetic resources, ITPGR apply to PGRFA, which is listed in the Annex. Since objectives of CBD are the conservation of biological diversity, the sustainable use of its components and the fair and equitable sharing of the benefits arising out of the utilization of genetic resources, it is associated with a variety of industrial sectors including agriculture, livestock industry, forestry, fisheries, and pharmaceutical industry, and it has a diverse set of stakeholders that need to be coordinated. Particularly in the sector of agriculture, some ABS issues are covered by the ITPGR and others by the CBD, depending on the kind and purpose of use of crop. This means that national focal point of ITPGR usually is appointed to the Ministry of Agriculture, but in the case of CBD, it will be Ministry of the Environment. In the case of Japan, toward the 10th Conference of Parties to the CBD, relevant ministries including Ministry of Agriculture and Ministry of the Environment decided to establish the “Meeting of relevant Vice-Ministers for CBD COP10” to discuss post-2010 targets, ABS, and the Cartagena Protocol (Ministry of the Environment, 2014). Therefore, it is considered that the issue of terminology is being coordinated among related ministries. Through the analysis of the views and negotiation above, in the middle of the negotiations, Japan made an argument that was different from its previously expressed position, but in the end, it became the same position as the previously expressed position. Spain, on the other hand, remains suspicious in relation to the EU. While the EU’s position indicated that GSD was the appropriate term, the Spanish government’s position was that the “Biological Database” was appropriate. The EU has submitted views only on the CBD, while Spain has submitted views only on the ITPGR. Since the focal point of the ITPGR in Spain is the Ministry of Agriculture, Fisheries and Food^34^, it may be probable that there was no coordination among the relevant ministries. In any case, whether it is a diplomatic strategy or the lack of government control is up to the parties concerned. Therefore, different authorities and related research communities may need to be involved, both nationally and internationally. The second is whether the term GSD is also used in CBD negotiation, as in the resolution on GLIS adopted at the Governing Body of ITPGR. On the other hand, as mentioned in the opinions and scoping studies from Germany^35^, the problem with the term is caused by the innovative technological development, and it is assumed that the term has no meaning even if it is defined in the future. Therefore, it is desirable to recognize uncertainties and continuously monitor technological development. In preparation for the 15th Conference of the Parties to the CBD, a group of technical experts called the “Multidisciplinary Technical Expert Group on Synthetic Biology” was proposed to monitor trends of such technology in discussions on synthetic biology^36^. In establishing such a group, it is necessary to consider relationships with existing institutions such as SBSTTA and securing funds, but this could be an alternative.

Uncertainties can arise not only from technological development and scientific perspective but also from institutional aspects. For example, if an issue is being discussed in the context of the implementation of the agreement, in relation to the article being discussed, and as a result, if a decision is taken as a resolution of the Conference of the Parties, or if the relationship between the resolution and the text of the agreement is not explicit, uncertainty may arise over the national response of the resolution^37^. As discussed at the ITPGR/GLIS, DSI/GSD is expected to be included in future development through discussions at expert meetings as it is included in data related to target plant genetic resources. GLIS has a role as MLS’s benefit-sharing [ITPGR Article 13 (2) (a)] and can be interpreted as having some bearing on the Board’s responsibilities, such as the development of policy guidelines and the adoption of recommendations. However, in the case of GISAID, which is not directly managed and operated by the Conference of the Parties, etc., prior coordination for decision-making at the Conference of the Parties is considered necessary in order to expect practical operation. Article 18 (3) of the CBD establishes the CBD Clearinghouse as a “mechanism for the exchange of information to promote and facilitate technical and scientific cooperation.” Therefore, in this context, it is considered that the establishment of an information sharing system is an option within the scope of authority delegated to the Conference of the Parties. However, as shown in the GLIS, there is a movement to seek cooperation with existing large-scale databases such as INSDC. For this reason, CBD is expected to cooperate with or improve existing mechanisms rather than building its own database.

## Conclusion

In addition to uncertainties over scientific knowledge, there were gaps in recognition of scientific knowledge among governments, and governments made efforts to reduce such uncertainties through preparatory meetings such as the Working Group and discussions at the Conference of the Parties and the Governing Body. However, such efforts have been dynamically developed not only into a single treaty but also into related treaties, and it is necessary to confirm the discussions comprehensively. Further, in the course of discussions, the term “information associated with PGRFA” was proposed, which is not based on scientific knowledge, but the term GSD was eventually used in the resolution of the GLIS without common understanding or interpretation. Decision-making varies from one agreement to another, but many of the MEAs discussed in this paper are based on consensus among the parties. Therefore, in order for scientific knowledge to be reflected in the decision making of the agreement, it is necessary to establish a governance that ensures mutual communication between the research community (regardless of field) and the governments of the Contracting Parties to the agreement to understand the various strategies through enhancements to existing systems or development of new mechanism such as VPM.

## Author Contributions

ED made a contribution based on his experience as an expert on the discussions at the GLIS under the ITPGR. KW has also contributed to the implementation of this research, including funding and general advice. All authors contributed to the article and approved the submitted version.

## Conflict of Interest

The authors declare that the research was conducted in the absence of any commercial or financial relationships that could be construed as a potential conflict of interest.

## Footnotes

1 Such obligations vary depending on the terms “as appropriate” and “when appropriate” and the interpretations of each country.

2 IISD. Summary of the seventh session of the Governing Body of the International Treaty on Plant Genetic Resources for Food and Agriculture. https://enb.iisd.org/download/pdf/enb09691e.pdf (accessed August 27, 2020).

3 ITPGR. Report of the Eighth Session of the Governing Body of the International Treaty on Plant Genetic Resources for Food and Agriculture, IT/GB-8/19/Report, http://www.fao.org/3/nb918en/nb918en.pdf (accessed August 27, 2020).

4 PIPF 5.2.

5 The report of the Review Group submitted to the 70th WHO General Assembly in April 2017 proposed including GSD in the definition of biological material, but it was not reflected in the General Assembly resolution [WHA 70 (10)].

6 ITPGR, Resolution 13/2017 (Multi-Year Programme of Work of the Governing Body of the International Treaty); CBD, CBD/NP/MOP/DEC/2/5 (2/5. Cooperation with other international organizations, conventions and initiatives); CBD/COP/DEC/14/20 (14/20. Digital sequence information on genetic resources).

7 Article 5.2.2 of PIPF recognizes GISAIDs as public-domain or public-access databases.

8 See https://www.gisaid.org/epiflu-applications/global-cases-covid-19/.

9 See http://www.insdc.org/policy.html.

10 Secretariat of CBD. Notification Digital Sequence Information on Genetic Resources, Ref.: SCBD/SPS/DC/VN/KG/jh/86500. https://www.cbd.int/doc/notifications/2017/ntf-2017-037-abs-en.pdf (accessed August 27, 2020).

11 Secretariat of CBD. Notification Digital Sequence Information on Genetic Resources: Submission of views and information and call for expression of interest to undertake studies, Ref.: SCBD/NPU/DC/VN/KG/RKi/87804. https://www.cbd.int/doc/notifications/2019/ntf-2019-012-abs-en.pdf (accessed August 27, 2020).

12 Secretariat of ITPGR. Notification Invitation to submit views and other information on Digital sequence information, NCP GB8-020 MYPoW/DSI. http://www.fao.org/3/ca4009en/ca4009en.pdf (accessed August 27, 2020).

13 Secretariat of ITPGR. Notification Invitation to submit views and other information on Digital sequence information, NCP GB8-016 MYPoW/DSI. http://www.fao.org/3/CA2701EN/ca2701en.pdf (accessed August 27, 2020).

14 Submissions are in the following: https://www.cbd.int/dsi-gr/2017-2018/#submissions, https://www.cbd.int/dsi-gr/2019-2020/submissions/ and http://www.fao.org/plant-treaty/overview/mypow/dsi/en/.

15 CBD. Digital sequence information on genetic resources, UNEP/CBD/COP/DEC/13/16. https://www.cbd.int/doc/decisions/cop-13/cop-13-dec-16-en.pdf (accessed August 27, 2020).

16 CBD. Digital sequence information on genetic resources, UNEP/CBD/COP/DEC/14/20. https://www.cbd.int/doc/decisions/cop-14/cop-14-dec-20-en.pdf (accessed August 27, 2020).

17 ITPGR. Multi-Year Programme of Work of the Governing Body of the International Treaty, IT/GB-7/17/Res13. http://www.fao.org/3/a-mv176e.pdf (accessed August 27, 2020).

18 IISD. Ninth Meeting of the Ad Hoc Open-ended Working Group to Enhance the Functioning of the Multilateral System of the International Treaty on Plant Genetic Resources for Food and Agriculture. https://enb.iisd.org/download/pdf/enb09731e.pdf (accessed August 27, 2020).

19 Secretariat of ITPGR. Enhancing the functioning of the Multilateral System: note by the Co-Chairs, IT/OWG-EFMLS-9/19/4. http://www.fao.org/3/ca5046en/ca5046en.pdf (accessed August 27, 2020).

20 IISD. Ninth Meeting of the Ad Hoc Open-ended Working Group to Enhance the Functioning of the Multilateral System of the International Treaty on Plant Genetic Resources for Food and Agriculture. https://enb.iisd.org/download/pdf/enb09731e.pdf (accessed August 27, 2020).

21 IISD. Ninth Meeting of the Ad Hoc Open-ended Working Group to Enhance the Functioning of the Multilateral System of the International Treaty on Plant Genetic Resources for Food and Agriculture. https://enb.iisd.org/download/pdf/enb09731e.pdf (accessed August 27, 2020).

22 ITPGR. The Vision and the Programme of Work on the Global Information System, IT/GB-6/15/Res 3. http://www.fao.org/3/a-bl140e.pdf (accessed August 27, 2020).

23 ITPGR. Implementation of the Global Information System, Resolution 4/2019. http://www.fao.org/3/nb782en/nb782en.pdf (accessed August 27, 2020).

24 ITPGR. The Vision and the Programme of Work on the Global Information System, IT/GB-6/15/Res 3. http://www.fao.org/3/a-bl140e.pdf (accessed August 27, 2020).

25 ITPGR. Third Meeting of the Scientific Advisory Committee on the Global Information System of Article 17 of the Treaty, IT/SAC-GLIS- 3/18/Report. http://www.fao.org/3/CA0526EN/ca0526en.pdf (accessed August 27, 2020).

26 ITPGR. Implementation of the Global Information System, Resolution 4/2019. http://www.fao.org/3/nb782en/nb782en.pdf (accessed August 27, 2020).

27 DAA is effective as of March 16, 2011.

28 Global Initiative on Sharing All Influenza Data (GISAID Initiative). Digital Sequence Information on Genetic Resources Submission by the GISAID Initiative. https://www.cbd.int/abs/DSI-views/GISAID-DSI.pdf (accessed August 27, 2020).

29 CBD. F. Terminology “indigenous peoples and local communities” in the Article 8(j) and related provisions, UNEP/CBD/COP/DEC/XII/12. https://www.cbd.int/doc/decisions/cop-12/cop-12-dec-12-en.pdf (accessed August 27, 2020).

30 CBD. Annex III of Report of the Twelfth meeting of the Conference of the Parties to the CBD, UNEP/CBD/COP/12/29. https://www.cbd.int/doc/meetings/cop/cop-12/official/cop-12-29-en.pdf (accessed August 27, 2020).

31 From the results of interviews with government officials who attended the informal meeting.

32 In the process of negotiating the Nagoya Protocol, developed countries such as Japan and the EU insisted on genetic resources covered by the treaty, while the African Group and Brazil insisted on including not only genetic resources but also their derivatives and products.

33 CBD apply to the benefits arising from the use of genetic resources, while countries such as the Philippines, Brazil, and South Africa apply biological resources and/or genetic information under their national ABS law.

34 See http://www.fao.org/plant-treaty/countries/national-focal-points/en/.

35 Germany. Submission of views and other information on “Digital sequence information”. http://www.fao.org/3/ca4237en/ca4237en.pdf (accessed August 27, 2020).

36 Secretariat of CBD. Synthetic Biology, CBD/SBSTTA/24/4. https://www.cbd.int/doc/c/5992/a98b/e60a7d8dde36888c553b92d4/sbstta-24-04-en.pdf (accessed August 27, 2020).

37 Although it is not the DSI, it was confirmed by the resolution of the Second Conference of the Parties to the CBD that human genetic resources are not included, but the ABS law of Malaysia, a party to the CBD, covers human genetic resources. At present, however, no issue has been raised by Malaysia regarding such measures at meetings of the Conference of the Parties, etc.
